# The Evolutionary Puzzle of Suicide

**DOI:** 10.3390/ijerph10126873

**Published:** 2013-12-09

**Authors:** Henri-Jean Aubin, Ivan Berlin, Charles Kornreich

**Affiliations:** 1 Hopital Paul Brousse, AP-HP, INSERM U 669, University Paris-Sud 11, 12 Avenue, Paul-Vaillant-Couturier, Villejuif 94800, France; 2 Hôpital Pitié-Salpêtrière, Assistance Publique-Hôpitaux de Paris, Université P&M Curie-Faculté de Médecine, INSERM U894, 47 Bd de l’Hôpital, Paris 75013, France; E-Mail: ivan.berlin@psl.aphp.fr; 3 Laboratoire de Psychologie Médicale, Université Libre de Bruxelles, CHU Brugmann, Place van Gehuchten 4, Bruxelles 1020, Belgium; E-Mail: ckornrei@ulb.ac.be

**Keywords:** suicide, ethology, genetics, depression, evolutionary psychology, Darwin

## Abstract

Mechanisms of self-destruction are difficult to reconcile with evolution’s first rule of thumb: survive and reproduce. However, evolutionary success ultimately depends on inclusive fitness. The altruistic suicide hypothesis posits that the presence of low reproductive potential and burdensomeness toward kin can increase the inclusive fitness payoff of self-removal. The bargaining hypothesis assumes that suicide attempts could function as an honest signal of need. The payoff may be positive if the suicidal person has a low reproductive potential. The parasite manipulation hypothesis is founded on the rodent—*Toxoplasma gondii* host-parasite model, in which the parasite induces a “suicidal” feline attraction that allows the parasite to complete its life cycle. Interestingly, latent infection by *T. gondii* has been shown to cause behavioral alterations in humans, including increased suicide attempts. Finally, we discuss how suicide risk factors can be understood as nonadaptive byproducts of evolved mechanisms that malfunction. Although most of the mechanisms proposed in this article are largely speculative, the hypotheses that we raise accept self-destructive behavior within the framework of evolutionary theory.

“*Natural selection will never produce in a being any structure more injurious than beneficial to that being, for natural selection acts solely by and for the good of each. No organ will be formed for the purpose of causing pain or for doing an injury to its possessor.*”

Charles Darwin

## 1. Introduction

Evolutionary theory is typically concerned with explaining life. Within this framework, selection is expected to promote the evolution of various biological mechanisms that increase the individual’s ability to avoid death. The evolution of mechanisms of self-destruction is more difficult to envision [1].

The estimated global burden of suicide is one million deaths per year, with a great inter-country variability [2]. Historically, suicide risk increased with age, with older men identified as the group at highest risk. However, in the 1970s, suicide became increasingly common in young adults, especially young men, in some high-income countries [3]. Distal risk factors include genetic loading, personality characteristics (e.g., impulsivity and aggression), restricted fetal growth and perinatal circumstances, early traumatic life events, and neurobiological disturbances. Proximal risk factors include psychiatric disorders, including substance use disorders, physical disorders, psychological crisis, availability of means, and exposure to suicidality models [2]. Heritability of completed suicide has been estimated at about 43% [4].

Most of the research on suicidal behavior has been conducted in an atheoretical context. However, diverse theories of suicide have been proposed. Biological theories suggest that suicidal behavior results from the dual presence of a biologically based vulnerability and an activating psychosocial stressor [5,6]. Psychodynamic theories propose that the suicidal crisis is the consequence of affect deluge, efforts to master affective flooding, loss of control and disintegration, and grandiose survival and body jettison [7]. Cognitive-behavioral theories posit causal roles of hopelessness, autobiographical memory deficits, perceptions of entrapment, and emotion dysregulation [8]. Developmental/systems theories posit causal roles of disturbed social forces and family systems [8]. The broader interpersonal theory of suicide proposes that the most dangerous form of suicidal desire depends on two constructs: (1) thwarted belongingness and (2) perceived burdensomeness. Suicide is likely to occur when the subject experiences hopelessness about these states and when he acquires the capability to engage in suicidal behavior [8]. Thwarted belongingness may result in self-defeating behaviors, perceptions of burdensomeness may lead to self-hate regarding those perceptions, and acquired capability is a multidimensional emergent variable that involves the dimensions of lowered fear of death and increased tolerance of physical pain.

Suicide and homosexuality are the two phenomena that have been most difficult to reconcile with evolutionary theory, as both directly affect human reproductive fitness [9,10]. Selection pressures modify physical characteristics and behavioral patterns to adapt to environmental changes and challenges. Evolutionary psychology has proposed that the brain was shaped by natural selection to solve adaptive problems that were repeatedly encountered by humans throughout their evolutionary history. The environment of evolutionary adaptedness refers to the problems hunter-gatherers had to solve and the conditions under which they solved them; the functional architecture of the mind was not selected for because it solved the problems faced in our modern environment, with its permanent settlements, agriculture, large interacting populations. Instead, it was shaped by how well it solved adaptive problems among our hunter-gatherers ancestors. Thus evolution by natural selection resulted in specialized neurocognitive mechanisms that successfully responded to distinct adaptive problems that occurred before the emergence of our modern environment [10,11]. Specialized mechanisms have been favored over more general mechanisms because they solve a number of problems that more generalized mechanisms cannot, e.g., functional incompatibility between two functions will favor specialized solutions rather than common general mechanism, combinatorial explosion paralyzes any truly domain-general system when encountering real-world complexity [12,13]. These specialized mechanisms have been referred to as “darwinian algorithms” [12]. Examples of specialized mechanisms designed for specific adaptive functions include fear of snakes and spiders, the capacity to learn spoken languages, preferences for particular characteristics in a mate, and the detection of cheaters [12].This theoretical framework contrasts with “blank slate” frameworks, which argue that a relatively few general-purpose mechanisms can account for humans’ reasoning and learning abilities [14].

Darwin postulated that variant traits are inherited by the offspring and that traits that are beneficial for survival and reproduction are transmitted to future generations at greater frequencies than alternatives. This process results in the following three products [10]: (a) adaptations, inherited traits that favor survival and reproduction; (b) byproducts, artifacts without adaptive value but that are inherently coupled with adaptations; and (c) noise, variations due to random environmental events or genetic mutations.

Evolutionary theorists stress the distinction between proximate and ultimate causation. Proximal causation explores how the mechanism works, whereas the higher-level ultimate causation explores why the phenomenon exists. The purpose of evolutionary psychology is to provide clues on ultimate causation. As natural selection favors genes that increase the ability to survive and reproduce, this mechanism should promote the emergence and maintenance of selfish behavior. However, humans are social animals that evolved with a high level of cooperation. Some situations present choices between alternatives that are beneficial to an individual and alternatives that are less beneficial—or even harmful—to the individual but would nevertheless be beneficial if chosen by many individuals. Such alternatives characterize problems of social cooperation; selection of the latter alternative is generally considered to be altruistic [15]. In other words, in an altruistic case, the altruistic subject experiences a fitness loss whereas the receiving subject benefits from a fitness gain.

Relatives often carry the same genes. Therefore, we can expect that a hypothetical gene that generates a behavior that increases the kin fitness at the expense the individual's own fitness will increase in frequency in the population. According to this fundamental principle of kin selection theory, at times, the fitness loss induced by the behavior can be compensated by an enhanced fitness of relatives. Kin selection is a special case of inclusive fitness, a more general principle that refers simply to the fact that identical gene copies are likely to be found in other subjects that are not necessarily kin [16]. Fitness criteria may show some degree of fluidity; for example, traits that have been transmitted through generations (with adaptive value in the ancestral environment) may lose their fitness importance in the modified modern environment. This phenomenon is called mismatch theory: traits that were adaptive in the ancestral ecosystem may not only use their adaptive value, but even have pathological consequence in the modern ecosystem [17] (for example an efficient energy storage system, essential in the ancestral environment, may lead to metobolic syndroms in the western modern environment). 

The purpose of this narrative review is to present several non-mutually exclusive hypotheses that propose adaptive mechanisms that lead to suicidal behavior.

## 2. The Altruistic Suicide Hypothesis

De Catanzaro developed a mathematical formula on the basis of the inclusive fitness theory and the concept of reproductive value to express the residual capacity of an individual to influence his inclusive fitness. This formula involves “the individual's remaining reproductive potential in his expected natural lifetime plus the summated impacts of his continued existence on the remaining reproductive potential of each of his kin, each weighted by the coefficient of relationship” [18]. This measure (*i.e.*, the residual capacity to influence ones inclusive fitness) is believed to predict the degree to which self-preservation is optimally expressed. For highly social species, such as humans, it has been shown that the association of burdensomeness toward kin and low residual reproductive potential can lead to negative values [18].

Altruistic self-destructive behavior has been found in a wide variety of organisms, ranging from unicellular organisms [1] and parasites [19] to social insects [20,21,22], and is debated in mammals [23]. Self-destructive defensive behavior has evolved independently in a number of social insect species in response to natural enemies. Self-sacrificing behavior has mainly been found in the context of nest protection [24]. Defensive self-sacrifice persists in social insects because of its positive impact on the fitness of the colony’s reproductive individuals. As with other forms of altruistic traits, the genes involved will pass on to subsequent generations. Social insects’ suicidal defense behaviors can be divided into the following three main categories [24]: (1) instantaneous defense—the enemy is actively engaged, with a fatal outcome for the defender; (2) preemptive suicidal defensive behavior—death is the price of the repair or occultation of the nest, that can occur preventively, even before the confrontation to the enemy; and (3) altruistic self-removal—sick and most likely infected individuals leave the nest, therefore reducing the risk of contaminating nestmates.

Although culture and pathology clearly bear on human suicide, some of its emotional concomitants could reflect an erosion of self-preservative genetic expression in conjunction with low reproductive potential and burdensomeness toward kin [25]. Social isolation and perceived burdensomeness toward family has been correlated with suicidal ideation [25]. Moreover, some subjects justify their suicidal behavior as a sacrifice to help their relatives [26,27]. Historically, suicide among Inuits was considered to be altruistic. Old or very ill adults would kill themselves or be killed, typically at their own request, during times of famine during poor hunting [28]. This differs from suicide martyrdom, such as performed by the kamikaze or the Hezbollah, which is believed to be primarily motivated by parochial altruism in the form of moral commitments to collective sacred values rather than by selective incentives such as financial compensation to their family [29,30]. It has been shown that parochial altruist groups have higher reproductive success than groups with fewer parochial altruists [31]. However, one could also hypothesize that in some cultural contexts, suicide martyrdom is the only avenue to suicide that does not damage afterlife prospects (*i.e.*, suicide is expressly forbidden in some religions) [32].

## 3. The Bargaining Hypothesis

It has been proposed that suicidal behaviors are costly cries for help that can be functional for those enmeshed in interpersonal conflicts [33,34]. It may be a warning strategy for subjects to signal to their social group in which they have a conflict that it may suffer costs if assistance or change is not provided [26]. It could function as an honest signal of need, motivating people with a pre-existing interest in helping an individual who honestly signals the need for help, but not those who falsely exaggerate their need [33]. In this view, suicide attempts are gambles. Needy individuals win if they survive and receive the help needed; however, they lose in the case of a fatal outcome. The suicidal signaling strategy could have evolved if the average benefits received by genes coding for the warning strategy exceed the average costs. Important for this hypothesis is the fact that most suicide attempts are nonfatal [35].

## 4. The Parasite Manipulation Hypothesis

Parasite-induced modifications of host behavior are known from a wide range of host-parasite associations [36]. In many cases, these behavioral changes are considered to be adaptive and benefit the parasite by increasing its probability of successful transmission [37]. Food chain transmission of parasites is typical of intermediate host manipulations, *i.e.*, the parasite is immature in the intermediate host; in order to attain maturity, and to achieve its life cycle, it must be ingested by a predatory definitive host [38,39].

Some parasites induce suicide-like behaviors in hosts [36]. *Schistocephalus solidus-*infected fish approach the water surface and are inattentive to predatory birds that serve as the parasite’s definitive hosts. A particular nematode must be transmitted from ants to fruit-eating birds; infected ants develop an inflated, red abdomen, looking like fruit, and perch motionlessly among red berries, waiting for predation by birds; terrestrial arthropods seek and fatally jump into water when infected by a nematode that needs to complete its cycle in aquatic worms. Such parasite manipulations engage the host in “suicidal” behaviors, for the sole parasite reproductive benefit.

The host-parasite system *Rattus novergicus-Toxoplasma gondii* provides an interesting model for a behavior manipulation that could affect humans. *T. gondii* is an intracellular protozoan that is capable of infecting all mammals. It has an indirect life cycle. The parasites definitive hosts are the felids (cat family); they are the only mammals emit *T. gondii* oocysts through their feces. When another mammal such as a wild rodent ingests oocysts, cysts grow in the intermediate host, most frequently in the brain, and are viable for the life of the host [38].

Several experimental studies have shown that *T. gondii* has evolved the capacity to turn the innate fear that rodents have of cats into a “suicidal” feline attraction. Whereas non-infected rats display a strong aversion to cat odor, infected rats are specifically attracted to cat odor [38,40,41,42]. In addition, *T. gondii* causes an increase in activity and a decrease in neophobic (the innate fear of novelty) and predator vigilance behavioral traits [43,44,45,46]. This is believed to increase the likelihood of the rodent being predated, thereby completing the parasite's life cycle. Other rodent fears do not seem to be altered [38,39,40,47]. Although *T. gondii* cysts are observed in a variety of brain regions within rats, the density of cysts may be significantly higher in the amygdala, a brain region that is important for both defensive behaviors and the conditioning of fear, and in the prefrontal cortex [42,48]. Changes in dopamine metabolism are believed to mediate the altered behavior [49]. Anti-*T. gondii* drugs and antipsychotic treatments of infected rats prevent host-specificity behavioral alterations from being displayed [40,41].

The prevalence levels of *T. gondii* infection are very high across species. For example, the prevalence is 56% in domestic and wildcats, 43% in lions, 20%–60% in wild rodents, and 13%–67% in wild birds [39,50]. In humans, the seroprevalence of *T. gondii* infection increases with age, does not vary greatly between sexes, and is lower in cold regions, hot and arid areas, or at high elevations. In general, incidence of the infection varies with the population group and geographic location. Seropositivity can be up to 75% by the fourth decade of life in El Salvador *vs.* an overall seroprevalence of 23% in the USA [51]. Primary infection with *T. gondii* is typically subclinical. Infection may however be seriously hazardous in immunocompromised patients or if acquired during pregnancy [52]. Mental disorders, mainly schizophrenia, have been related to latent infection with *T. gondii* [53,54,55,56,57]. Behavioral alterations (often subtle) that are similar to those observed in *T. gondii*-infected rodents have been observed in latently infected humans, such as increased activity, decreased reaction times and altered personality profiles [39,58,59].

Latent infection by *T. gondii* has been linked to a higher risk of suicidal behavior in humans [57,60,61,62,63,64,65]. *T. gondii* infection is also linked to a significantly increased risk of car accidents [66]. Interestingly, it has been reported that more than 2% of traffic accidents are suicidal behaviors [67]. Large felids (tigers, leopards, lions) were the primary mammalian predators of humans in the 20th century [68,69], and these species (along with other large felids that co-existed in our ancestral environment) are likely to have been significant predators of early humans. The inference that *T. gondii* manipulates human behavior as it manipulates rodents to increase the likelihood to being predated by its definitive host is speculative. However, we have displayed a set of arguments that lead to the hypothesis that *T. gondii* may manipulate human behavior to increase suicidality, thus serving an evolutionary purpose (Table 1). 

## 5. Suicide Risk Factors in Light of Evolutionary Psychology

Although burdensomeness to kin may provide a plausible explanation for some suicides among the elderly, it could be more difficult to accept that suicide may enhance the fitness of relatives in the case of a healthy adolescent. Instead, such suicides are likely to be non adaptive byproducts of evolved mechanisms that malfunction [10].

### 5.1. Psychiatric Disorders

Psychological autopsies of suicide have shown that a context of mental disorder is present in the vast majority of cases. Some aversive states are the product of natural selection. For instance, pain, fever, nausea are known to be useful responses in some circumstances. Likewise, the aversiveness of negative emotional states can also be useful. Many adaptive functions have been suggested for low mood or depression: facilitating disengagement from unreachable responsibility, expressing a need for help, and signaling the act of yielding in a hierarchy conflict [70]. An increased fitness may result from sadness, pessimism and lack of motivation, in the way that they lead to the inhibition of certain behaviors, for example actions in the absence of a practicable plan, dangerous or futile challenges to dominant figures, efforts that would eventually damage the organism [70]. Crying elicits empathy, comforting behaviors in observers and may help strengthen bonds and result in an increase in social support. Feelings of worthlessness and guilt might motivate introspective understanding of how its actions were problematic and may also elicit forgiveness from others involved in the situation [71].

Prominent cognitive theories propose that negative cognitive biases, characterized by a tendency to preferentially process negatively valenced information, play a central role in depression [72]. Clinical and subclinical levels of depression are associated with difficulty in disengaging attention from negative self-relevant stimuli and attentional avoidance of positive stimuli [73]. Thus, although depression may have adaptive features in some circumstances, the negative cognitive bias that accompanies this condition is likely to result in an overvaluation of one’s perceived burdensomeness and, therefore, increase the risk of altruistic suicidal behavior (Table 1).

Even without the specific cognitive bias found in depressive disorders, the emotional distress that accompanies most psychiatric disorders is likely to increase perceived burdensomeness to some extent.

**Table 1:** Whose genes may benefit from the suicidal behavior?

**Suicidal Behavior Type**	**Ancestral Ecosystem**	**Western Modern Ecosystem**
**Physical Disorders**	Kin’s genes could benefit from the suicidal behavior of an individual imposing high burdensomeness and having low reproductive potential	As burdensomeness is shared by social welfare, virtually no genes benefit from the suicidal behavior
**Depression**	No genes directly benefit from the suicidal behavior. Suicide is a by-product of negative cognitive bias (overevaluation of perceived burdensomeness)	No genes directly benefit from the suicidal behavior: no significant change in our current environment
**Suicide Martyrdom**	Ethnic group genes could benefit from a parochial altruistic suicidal behavior	Ethnic group genes: no significant change in our current environment
**Bargaining Suicidal Behavior**	The suicidal individual’s genes could benefit if the suicide fails and the help needed provided	The suicidal individual’s genes: no significant change in our current environment
**Males Impulsive Suicidal Behavior**	No genes directly benefit from the suicidal behavior. Suicide is a by-product of risk-taking	No genes directly benefit from the suicidal behavior: however risk-taking is likely to be less beneficial to the genes involved in the western modern than in the ancestral ecosystem
**Parasite Manipulation**	*T. gondii* genes benefit from the suicidal behavior	No genes benefit anymore from the suicidal behavior (some exceptions might occur in India and Africa *)

Note: * Large felids (tigers, leopards, lions) were the primary mammalian predators of humans in the 20th century, especially in India and Africa [68].

### 5.2. Physical Disorders

Severe physical disorders are obvious conditions in which burdensomeness is increased, especially when the disorders are chronic and disabling. According to the pathogen host defense theory, risk alleles for depression originated and have been retained in the human genome because these alleles promote pathogen host defense, which includes a suite of immunological and behavioral responses to infection [74]. One of these responses could be self-removal from kin to prevent the transmission of infection [75] (Table 1).This phenomenon has been observed in other social animals [20,24].

### 5.3. Gender

One of the most robust patterns in suicide is that men commit suicide more frequently than do women [2,3]. Sexual selection and the subsequent differential parental investment of the two sexes have shaped the manner in which men and women engage in intra-sexual competitions within the mating arena. Given that men face greater variance in reproductive fitness, they have evolved a strong penchant for risk-taking across numerous domains, such as physical and financial domains. Risk-taking can be construed as an adaptive response to the stronger sexual selection pressures faced by men [9]. Risk-taking is supported by some personality traits such as impulsivity-aggressiveness, which predisposes individuals to suicide, especially young men [76]. Impulsivity, which is believed to enhance inclusive fitness by increasing readiness to fight or flee in dangerous circumstances [26], may have been adaptive in the environment of evolutionary adaptedness [77,78] (Table 1). Compared to women, the stronger sexual selection pressures faced by men yield a more pronounced assortment of “winners” and “losers” in the mating field. Accordingly, some suicides can be conceived as the most extreme form of defeatism in the context of mating competition [9]. Men are more likely to commit suicide as a result of loss of status (employment, wealth, marriage), whereas women are more likely to do so because of romantic, emotive or familial concerns [79,80]. It has been hypothesized that social defeats that result in one’s degradation within the relevant dominance hierarchy can be sufficiently injurious to lead to suicide [9]. Interestingly, it has been recently shown that local sex ratio, a proxy for mating market conditions, is strongly linked to male suicide [80].

## 6. Conclusions

Although not all of the accumulated findings on suicide are within the purview of evolutionary theory, many are congruent with Darwinian-based expectations. We have shown that some features are compatible with the view that suicide can have adaptive values. In particular, the altruistic suicide hypothesis proposes that when both poor reproductive potential and perceived burdensomeness are present, self-removal can increase inclusive fitness. This ultimate explanation of increased suicide rates observed with senescence, psychiatric disorders, physical disorders, and homosexuality is compatible with the interpersonal theory of suicide proposed by van Orden *et al.* [8]. This theory, with no reference to evolutionary mechanisms, postulates that suicidal desire is particularly hazardous when caused by the simultaneous presence of two interpersonal constructs—thwarted belongingness and perceived burdensomeness. Our evolutionary views harbours the cognitive-behavioral interventions based on this model, e.g., restructuring of irrational negative beliefs and cognitive distortions related to burdensomeness over-perception, social skills training to decrease isolation. The bargaining hypothesis of suicide also proposes an adaptive value of suicidal behavior. Interestingly, in some circumstances, suicidal behavior may not be adaptive for the subject displaying suicidal behavior, but for the parasite it hosts. The higher suicide rate observed in males can be understood as a byproduct of mating competition that results in high-risk taking behavior and the struggle to increase one’s social status.

Our economic and cultural landscape has evolved exponentially. Individuals cooperate on an increasing scale and rely less on kin than in the past. We believe that the social welfare developed in many countries dramatically altered the adaptive value of suicidal behavior, which can be understood today as a mismatch: following our environmental change, the adaptive value of suicide has mainly been lost (Table 1).

In his *Letter to the American People*, the Surgeon General David Satcher asserted that suicide should be viewed as a serious public health problem [81]. Prevention efforts should be reinforced at the population level and in high-risk groups. Unless psychological models of human health behavior are infused with the evolutionary paradigm, they may risk the continued delivery of programs that offer only brief, suboptimal accounts of its causes [82]. A deeper understanding of the adaptive significance of suicide could aid in improving prevention strategies, notably through reducing perceived burdensomeness and perceived defeatism in the mating arena.
